# Common complement factor H polymorphisms are linked with periodontitis in elderly patients

**DOI:** 10.1002/JPER.22-0005

**Published:** 2022-05-04

**Authors:** Aino Salminen, Milla Pietiäinen, Susanna Paju, Timo Sorsa, Päivi Mäntylä, Kåre Buhlin, Juha Sinisalo, Pirkko J. Pussinen

**Affiliations:** ^1^ Oral and Maxillofacial Diseases University of Helsinki and Helsinki University Hospital Helsinki Finland; ^2^ Institute of Dentistry University of Eastern Finland Kuopio Finland; ^3^ Oral and Maxillofacial Diseases Kuopio University Hospital Kuopio Finland; ^4^ Division of Periodontology Department of Dental Medicine Karolinska Institutet Huddinge Sweden; ^5^ Heart and Lung Center Helsinki University Hospital Helsinki Finland

**Keywords:** genetics, inflammation, periodontitis, saliva

## Abstract

**Background:**

In our recent genome‐wide association study, we found that genetic polymorphisms in the complement factor H (CFH) gene and S100A gene region are strongly associated with serum matrix metalloproteinase 8 (MMP‐8) concentration and the release of MMP‐8 from neutrophils. As MMP‐8 is centrally involved in the pathogenesis of periodontitis, we aimed to evaluate the presence of genetic polymorphisms of *S100A8/A9/A12*, *MMP8*, and *CFH* in periodontitis. In addition, we studied whether polymorphisms of these genes affect the concentrations of S100A8, S100A12, MMP‐8, or complement activation marker in saliva.

**Methods:**

We genotyped four single‐nucleotide polymorphisms (SNPs, rs1560833 in *S100A8/A9/A12*, rs11225395 in *MMP8*, rs800292 in *CFH*, and rs1061170 in *CFH*) and measured salivary concentrations of S100A8, S100A12, MMP‐8, and terminal complement complex (TCC) in the Parogene cohort (n = 508). The cohort was composed of patients with an indication to coronary angiography and all underwent a clinical and radiographic oral examination.

**Results:**

*CFH* polymorphisms rs800292 and rs1061170 were associated with periodontal parameters. None of the polymorphisms showed association with salivary proteins. However, salivary concentrations of S100A8, S100A12, MMP‐8, and TCC were strongly associated with the number of periodontal pockets and alveolar bone loss.

**Conclusion:**

Interestingly, genetic variants of *CFH*, *MMP8*, and *S100A8/A9/A12* gene regions did not affect salivary levels of measured proteins. However, saliva levels of S100A8, S100A12, MMP‐8, and TCC, and *CFH* polymorphisms were associated with clinical and radiographic signs of periodontitis. Our study further supports the observations that any dysregulation of complement may increase the risk of inflammatory disorders, such as periodontitis.

## 1 INTRODUCTION

Periodontitis is initiated by disturbances in the dental biofilm and host homeostasis.^1^ The host response against the microorganisms leads to local and systemic inflammation, and, eventually, to degradation of the tissues surrounding teeth. The dysregulation of the host response by periodontal bacteria leads to destructive inflammation, which results in the symptoms of periodontitis such as the formation of periodontal pockets.^1^

The complement system is a network of interacting molecules that trigger, amplify, and regulate the immune and inflammatory response.^2^ Dysregulation of the complement system can destabilize host‐microbe homeostasis and cause inflammatory tissue damage.^1^, ^2^ Evidence from numerous studies shows that the activation of complement is implicated in the pathogenesis of periodontitis.^1^ Continuous complement activation and modulation by bacteria within the biofilm may enhance local tissue destruction, leading to formation of periodontal pockets, alveolar bone loss (ABL), and, eventually, loss of teeth.

Complement factor H (CFH) is an important inhibitory regulator of the alternative pathway of complement.^3^ Several genetic polymorphisms have been detected in the gene of CFH. Two single‐nucleotide polymorphisms (SNPs), rs800292 and rs1061170, are extensively studied since they lead to changes in the amino acid sequence of the CFH protein and have functional consequences.^4^, ^5^ The prevalence of the minor alleles of these SNPs in a Finnish population were 30% for rs800292 and 43% for rs1061170, making them relatively common.^6^ Both SNPs are strongly associated with the risk of age‐related macular disease (AMD), which is an inflammatory disease characterized by complement hyperactivity and tissue destruction.^4^, ^7^ The effect of these polymorphisms on other destructive inflammatory diseases, such as periodontitis, is not known.

S100 calcium‐binding proteins A8, A9, and A12 (S100A8, S100A9, and S100A12) are important proinflammatory mediators expressed mainly by neutrophils in various inflammatory reactions.^8^, ^9^ They activate Toll‐like receptors, induce cytokine secretion, and enhance particularly the innate immune response.^8^, ^9^ In recent years, saliva S100A8 and S100A9 have been shown to be promising biomarkers for periodontitis.^10^, ^11^, ^12^ In addition to saliva, also systemic concentration of the S100A8/A9 complex (calprotectin) was higher in aggressive periodontitis patients.^11^, ^13^ Polymorphisms in S100A8 gene were also associated with aggressive periodontitis.^13^ According to our knowledge, there are no other previous studies on the association between genetic polymorphisms of *S100A8*, *S100A9*, or *S100A12* and periodontitis.

The terminal complement complex (TCC), also known as membrane attack complex (MAC) or terminal C5b‐9 complex, is composed of proteins that assemble on the cell membranes of pathogens as a result of the activation of the complement system. Assembly of the TCC leads to pores that disrupt the cell membrane of target cells, leading to pathogen cell lysis and death. TCC concentration reflects the activation of complement, irrespective of the initial complement pathway involved; therefore, it is suitable for general evaluation of complement activation. When the basic MAC structure associates with regulatory proteins, its insertion into lipid bilayers is inhibited and soluble MAC is formed.^14^ Soluble MAC can be measured from plasma, urine, cerebrospinal fluid, or joint fluid.

Enhanced expression and activation of matrix metalloproteinase 8 (MMP‐8) is one of the key factors responsible for tissue degradation during periodontitis.^15^ Elevated levels of MMP‐8 are found in the saliva and gingival crevicular fluid as well as in the circulation of periodontitis patients.^15^ We recently found in our genome‐wide association study that genetic polymorphisms in the *CFH* gene and in *S100A8*/*S100A9/S100A12* gene region are strongly associated with serum MMP‐8 concentrations.^6^ These polymorphisms lead to enhanced release of MMP‐8 from neutrophils.^6^ Moreover, polymorphism in the promoter region of the *MMP8* gene is associated with the expression of *MMP8*.^16^ As MMP‐8 is a central mediator of tissue destruction in periodontitis, genetic polymorphisms that affect its gene expression or its release from the cells might affect the individual's susceptibility to periodontal disease.

The aim of our study was to evaluate the presence of *S100A8/A9/A12*, *MMP8*, and *CFH* polymorphisms in periodontitis and to find out if polymorphisms of these genes affect the concentrations of corresponding proteins or marker of complement activation in saliva.

## 2 MATERIALS AND METHODS

### 2.1 Study subjects

The details of the Parogene study cohort, sample size estimation, and inclusion and exclusion criteria have been described earlier.^17^ The general purpose of the Parogene study was to investigate the association between cardiovascular disease and oral health concentrating on periodontitis.^18^ The Parogene study was a substudy of the Corogene study. The Corogene study was conducted in Helsinki University Hospital and included 5295 symptomatic Finnish patients assigned to undergo coronary angiography.^17^ Blood samples were drawn from the arterial line during the angiogram and serum was prepared according to the laboratory standards of the Helsinki University Hospital. All study subjects signed an informed consent form, and the study was approved by the ethics committee of the Helsinki University Hospital (426/E5/05, 205/E0/2007, 52/2016, 1203/2016, and 187/E5/07).

### 2.2 Oral examination

Ten percent of the Corogene study participants were randomly selected to be invited to participate in the Parogene study. Parogene subjects were examined by periodontal specialists. The examination included a detailed clinical and radiographic oral examination and saliva sample collection. Probing pocket depths (PPDs) were measured from six sites of each tooth and bleeding on probing (BOP) and suppuration were registered from four sites of each tooth. In addition, furcation lesions, mobile teeth, mucosal findings, and caries were registered. The extent of ABL was evaluated from panoramic radiographs as described earlier and categorized into no, mild, moderate, severe, or total bone loss.^18^ The dentate subjects were divided into two groups based on their periodontal status: 346 subjects with no or mild periodontitis (<4 sites with PPD of ≥4 mm, no ABL or mild ABL) and 131 subjects with moderate to severe periodontitis (at least four sites with PPD of ≥4 mm and ABL from moderate to severe). In addition, there were 30 edentulous subjects. The total number of subjects available for the study is 507.

### 2.3 Saliva sample collection

At the beginning of the oral examination, the participants chewed a piece of paraffin for 5 minutes and at least 2 mL of stimulated whole saliva was collected. The samples were stored at −70°C until analyses.

### 2.4 DNA isolation and genotyping

DNA used in SNP genotyping was previously extracted from the saliva pellets.^19^ SNP analyses were performed with TaqMan SNP genotyping assays^1^. Reactions contained 1x TaqMan genotyping master mix^2^, 1x Taqman genotyping assay, and 20 ng template DNA in the total volume of 25 μL. Reactions were run according to manufacturer's instructions with a qualitative polymerase chain reaction (qPCR) system^3^.

Rs1061170 genotyping was performed with conventional PCR and restriction analysis. PCR reactions contained 500 nM primers (forward primer: 5′‐CCTTTGTTAGTAACTTTAGTTCGTCTT‐3′, reverse primer: 5′‐CCAAAAACTAAATAGGTCCATTGGT‐3′), 1x PCR master mix^4^, and 1 μL of template DNA. Total volume of the reactions was 20 μL. Thermal protocol included following steps: initial denaturation 98°C 30 seconds, denaturation 98°C 10 seconds, annealing 63.5°C 10 seconds, extension 72°C 15 seconds (30 cycles), and final extension 72°C 5 minutes. For restriction enzyme digestions, 10 μL of 1x green buffer containing 0.8 μL *Tas*I enzyme^5^ was added to the PCR reactions. Samples were incubated 10 minutes in 65°C and run on 2% agarose gel. The 500 bp PCR product with allele T had four restriction sites and allele C three restriction sites for *Tas*I.

### 2.5 Analyses of salivary S100A8, S100A12, TCC, and MMP‐8

Concentrations of S100A8, S100A12, and TCC were determined from thawed saliva samples by commercial enzyme‐linked immunosorbent assay (ELISA) kits^6^^,^ 7 ^,^ 8 according to the manufacturers’ instructions. Saliva MMP‐8 concentration was measured with a time‐resolved immunofluorometric assay (IFMA) in our previous study.^20^

### 2.6 Statistical analysis

The concentrations of salivary proteins were logarithmically transformed as they had skewed distributions. For determining high BOP levels and high periodontal pocket numbers, the subjects with BOP or more than zero pockets were divided into tertiles. The highest tertile was used as the test group and the two lowest tertiles as the reference group. For BOP, the cutoff value was 44%, for the number of pockets with PPD 4 to 5 mm the cutoff was 17 pockets, and for the number of pockets with PPD ≥6 mm the cutoff was seven pockets. Concentrations of proteins were compared between subgroups of patients divided according to periodontal parameters, smoking status, diabetes, and coronary artery disease (CAD) status by Kruskal‐Wallis test. Frequencies of periodontal conditions, diabetes, and CAD were compared between genotype groups by chi‐squared test. Association between SNP genotypes and periodontal parameters were analyzed by logistic regression adjusted for age, sex, smoking, diabetes, and the number of teeth. Edentulous individuals were excluded from the analyses of periodontal parameters. A similar logistic model was used to analyze the association between periodontal parameters and salivary concentrations of S100A8, S100A12, and TCC. Associations between SNP genotypes and salivary protein concentrations were analyzed with linear regression modeling adjusted for age, sex, smoking, diabetes, and the number of teeth. An additive model for SNP genotypes was used in all regression models.

## 3 RESULTS

Characteristics of the periodontitis groups (no to mild and moderate to severe periodontitis) are presented in Table 1. Twenty‐seven percent of the participants had moderate to severe periodontitis. The mean age of the study sample was 63 years, and 65% of them were male. In the moderate to severe periodontitis group, the mean age was slightly higher than in the no to mild periodontitis group. The mean number of teeth was 23 in the no to mild periodontitis group and 18 in the moderate to severe periodontitis group. Seventy percent of the participants had CAD, and 24% had diabetes. Periodontal findings were also frequent. Mild ABL was found in 45% of participants, while severe bone loss was rare (5.7%). The mean number of periodontal pockets with PPD 4 to 5 mm was 13. Deeper pockets were not as frequent: most of the participants did not have pockets with PPD ≥ 6 mm. However, some participants had high numbers of deep pockets (PPD ≥ 6 mm) and the maximum number detected in one individual was 70. As expected, the subjects with moderate to severe periodontitis had more BOP than those with no to mild periodontitis.

**TABLE 1:** Characteristics of the study sample

	No – mild periodontitis	Moderate – severe periodontitis
	**N (%)**	
Sex		
Male	215 (61.8)	98 (74.8)
Female	132 (38.2)	33 (25.2)
Smoking		
No	188 (54.3)	40 (30.5)
Ex	130 (37.6)	62 (47.3)
Current	28 (8.1)	29 (22.1)
CAD		
No	116 (33.5)	28 (21.4)
Yes	227 (66.2)	103 (78.6)
Diabetes		
No	269 (77.7)	94 (71.8)
Yes	70 (20.2)	37 (28.2)
Alveolar bone loss		
No	114 (32.9)	0
Mild	215 (62.1)	0
Moderate	14 (4.0)	107 (81.7)
Severe‐total	3 (0.9)	24 (18.3)
	**Mean (SD)**	
Age (years)	62.1 (9.60)	65.4 (7.48)
BMI (kg/m^2^)	28.0 (5.09)	27.6 (5.24)
Number of teeth and implants	22.6 (7.11)	18.1 (7.50)
BOP (%)	33.6 (16.4)	47.5 (32.4)
Number of pockets with PPD 4 to 5 mm	10.4 (10.9)	21.2 (15.3)
Number of pockets with PPD ≥6 mm	1.11 (2.71)	9.44 (13.6)

Abbreviations: BMI, body mass index; BOP, bleeding on probing; CAD, coronary artery disease; PPD, probing pocket depth; SD, standard deviation.

Minor allele frequencies of the SNPs were 0.29 for rs1560833 (*S100A9*), 0.42 for rs11225395 (*MMP8*), 0.28 for rs800292 (*CFH*), and 0.44 for rs1061170 (*CFH*) (Table 2).

**TABLE 2:** Characteristics of the SNPs

rs ID	Nearest gene	Type	Major/minor allele	MAF
rs1560833	*S100A9*	Intergenic	G/A	0.29
rs11225395	*MMP8*	Promoter	G/A	0.42
rs800292	*CFH*	Exon; missense	G/A	0.28
rs1061170	*CFH*	Exon; missense	T/C	0.44

Abbreviations: CFH, complement factor H; MAF, minor allele frequency; MMP, matrix metalloproteinase; SNP, single‐nucleotide polymorphism.

Table 3 displays the frequencies of ABL (moderate to severe), high BOP, high number of periodontal pockets (with PPD 4 to 5 mm or PPD ≥ 6 mm), periodontitis, diabetes, and CAD in groups of individuals with different SNP genotypes. Individuals with genotype GA for rs11225395 had less frequently pockets with PPD 4 to 5 mm and lower BOP% than those with genotypes GG or AA. The individuals with rs800292 genotype AA (two minor alleles) had less frequently ABL (14%) than those with genotypes GG (34%) or GA (29%). The individuals with rs1061170 genotype CC (two minor alleles) had fewer periodontal pockets with PPD 4 to 5 mm (18% had more than 17 pockets with PPD 4 to 5 mm) than those with genotypes TT (35% having high number of pockets) or CT (30% having high number of pockets).

**TABLE 3:** Frequencies of periodontal conditions, CAD, and diabetes in individuals with different genotypes of rs1560833, rs11225395, rs800292, and rs1061170

		ABL moderate‐severe	PPD4 ≥ 17*	PPD6 ≥ 7^†^	BOP > 44%	Periodontitis	CAD	Diabetes
		N (%)						
rs1560833	GG	74 (31%)	66 (27%)	31 (13%)	74 (30%)	65 (28%)	175 (70%)	58 (24%)
	GA	57 (31%)	60 (32%)	28 (15%)	65 (33%)	51 (28%)	145 (74%)	46 (24%)
	AA	10 (25%)	9 (23%)	5 (13%)	11 (26%)	8 (21%)	24 (57%)	10 (24%)
		*P* = 0.72	*P* = 0.42	*P* = 0.84	*P* = 0.58	*P* = 0.64	*P* = 0.10	*P* = 0.99
rs11225395	GG	46 (31%)	54 (36%)	18 (12%)	39 (25%)	42 (28%)	121 (77%)	40 (26%)
	GA	80 (33%)	57 (23%)	36 (15%)	93 (36%)	69 (29%)	174 (68%)	63 (25%)
	AA	15 (21%)	24 (33%)	10 (14%)	18 (24%)	13 (18%)	49 (65%)	11 (15%)
		*P* = 0.13	***P* = 0.02**	*P* = 0.76	***P* = 0.02**	*P* = 0.20	*P* = 0.08	*P* = 0.14
rs800292	GG	83 (34%)	70 (29%)	38 (16%)	73 (30%)	72 (30%)	178 (69%)	62 (24%)
	GA	52 (29%)	47 (26%)	23 (13%)	68 (38%)	46 (26%)	132 (71%)	38 (21%)
	AA	6 (14%)	17 (41%)	3 (7%)	9 (21%)	6 (14%)	33 (77%)	14 (33%)
		***P* = 0.03**	*P* = 0.19	*P* = 0.30	*P* = 0.06	*P* = 0.09	*P* = 0.55	*P* = 0.25
rs1061170	TT	45 (30%)	53 (35%)	19 (13%)	42 (27%)	40 (27%)	106 (68%)	43 (28%)
	TC	65 (30%)	65 (30%)	36 (16%)	74 (32%)	59 (27%)	167 (73%)	49 (22%)
	CC	28 (31%)	17 (18%)	8 (9%)	32 (32%)	23 (26%)	65 (66%)	21 (21%)
		*P* = 0.99	***P* = 0.02**	*P* = 0.16	*P* = 0.53	*P* = 0.96	*P* = 0.33	*P* = 0.29

*Note*: Chi‐squared test. Statistically significant *P*‐values are indicated by boldface.

Abbreviations: ABL, alveolar bone loss; BOP, bleeding on probing; CAD, coronary artery disease; PPD, probing pocket depth.

^*^Number of pockets with PPD 4 to 5 mm ≥ 17.

^†^Number of pockets with PPD **≥** 6 mm ≥ 7.

**FIGURE 1:**
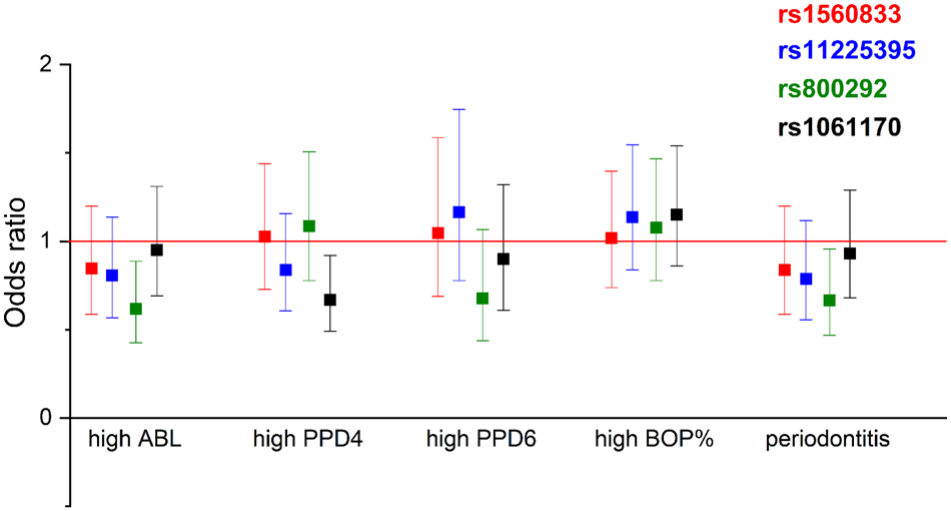
Associations between SNPs and periodontal parameters. Logistic regression adjusted for age, sex, smoking, diabetes, and the number of teeth. Additive model for SNP genotype. Error bars represent 95% confidence intervals for odds ratios. High ABL: ABL from moderate to severe; high PPD4: ≥ 17 sites with PPD 4 to 5 mm; high PPD6: ≥ 7 sites with PPD ≥ 6 mm; high BOP: BOP% ≥ 44. ABL, alveolar bone loss; BOP, bleeding on probing; PPD, probing pocket depth; SNP, single‐nucleotide polymorphism

The minor allele of rs800292 (*CFH*) was associated with lower odds of having moderate – severe periodontitis (odds ratio [OR], 0.67; 95% confidence interval [CI], 0.47 to 0.96, *P* = 0.03; Figure 1) and ABL (OR, 0.62; 95% CI, 0.43 to 0.89; *P* = 0.01). The minor allele of the other *CFH* SNP, rs1061170, was also associated with a lower number of pockets with PPD 4 to 5 mm (OR, 0.67; 95% CI, 0.49 to 0.92; *P* = 0.01). Rs1560833 (*S100A9*) or rs11225395 (*MMP8*) were not associated with any periodontal parameters.

**FIGURE 2:**
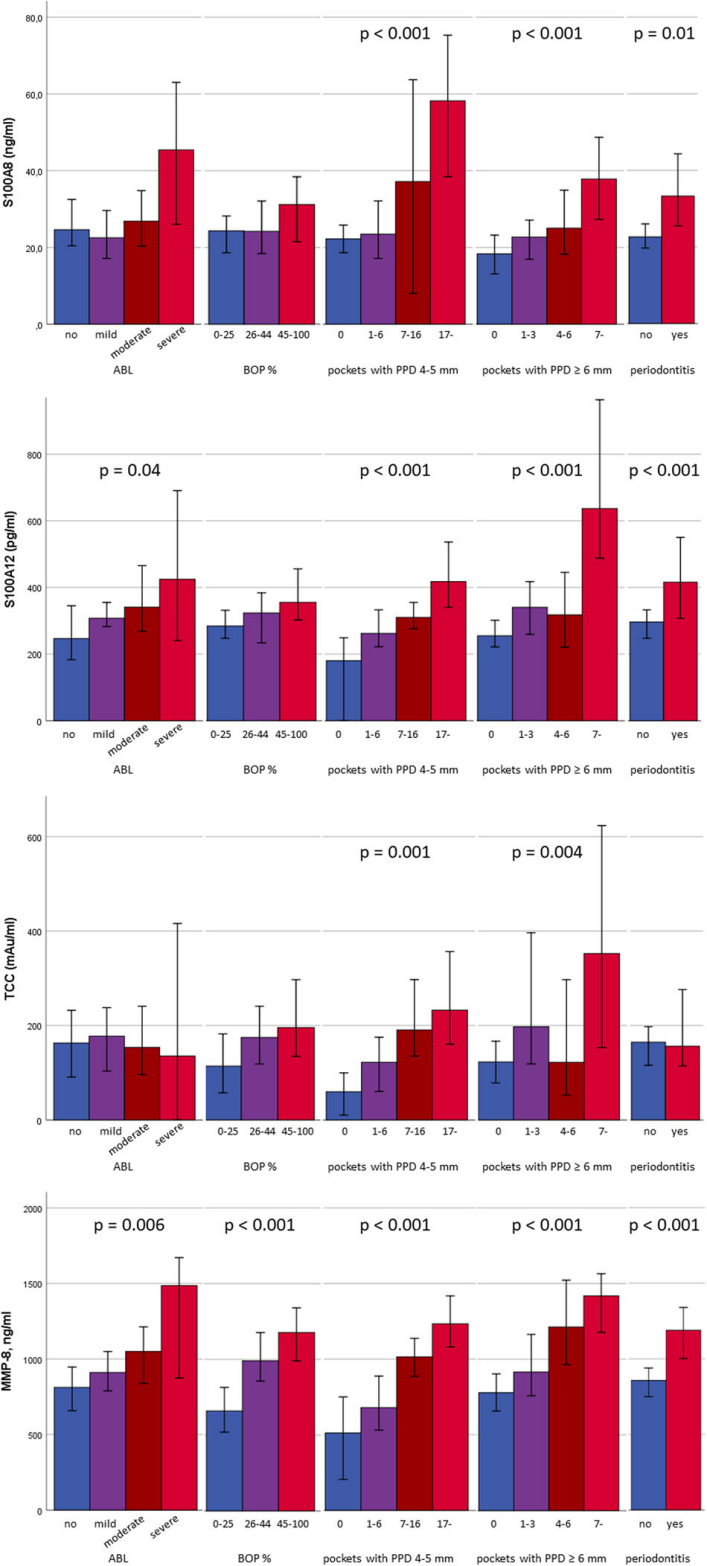
Concentrations of salivary proteins in subgroups of study participants. Concentrations are expressed as medians. Error bars represent 95% confidence intervals. Comparisons between the groups were made by the Kruskal‐Wallis test

The median concentrations of salivary proteins in the subgroups of the study subjects divided according to periodontal parameters are presented in Figure 2. Salivary concentrations of S100A8, S100A12, MMP‐8, and TCC increased along with increasing numbers of periodontal pockets with PPD 4 to 5 mm or PPD ≥ 6 mm (Figure 2). Concentrations of S100A8, S100A9, or TCC did not differ between subgroups of patients divided according to BOP levels, but the concentration of MMP‐8 was significantly higher in those with high BOP. S100A12 and MMP‐8 concentrations were higher in patients with ABL, while S100A8 and TCC did not differ between ABL subgroups.

Salivary TCC concentration was significantly lower in smokers compared to nonsmokers (see Supplementary Table S1, which is available in the online *Journal of Periodontology*). The concentrations of S100A8 and S100A12 in saliva were significantly higher in diabetics compared to nondiabetics. In CAD patients, concentrations did not differ from controls.

**FIGURE 3:**
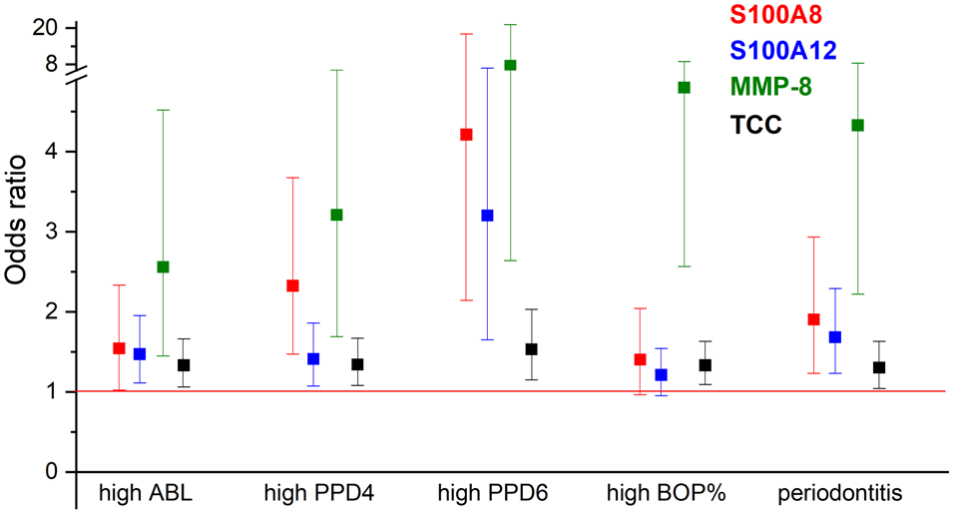
Associations of salivary S100A8, S100A12, MMP‐8, and TCC concentrations with periodontal parameters. Odds ratio for 10‐fold increase in concentration. Logistic regression adjusted for age, sex, smoking, diabetes, and the number of teeth. Error bars represent 95% confidence intervals for odds ratios. High ABL: ABL from moderate to severe; high PPD4: ≥ 17 sites with PPD 4 to 5 mm; high PPD6: ≥ 7 sites with PPD ≥ 6 mm; high BOP: BOP% ≥ 44. ABL, alveolar bone loss; BOP, bleeding on probing; MMP‐8, matrix metalloproteinase 8; OR, odds ratio; PPD, probing pocket depth; TCC, terminal complement complex

Associations between salivary proteins and periodontal parameters are shown in Figure 3. Salivary S100A8, S100A12, and MMP‐8 concentrations were associated with periodontitis, ABL, the number of periodontal pockets with PPD 4 to 5 mm, and especially the number of periodontal pockets with PPD ≥ 6 mm (OR for 10‐fold increase in S100A8: 4.22; 95% CI, 2.15 to 8.30; *P* < 0.001). Salivary TCC concentration was associated with periodontitis, ABL, BOP, the number of periodontal pockets with PPD 4 to 5 mm, and the number of periodontal pockets with PPD ≥ 6 mm. There was no association between SNP genotypes and salivary protein concentrations (see Supplementary Table S2 in the online *Journal of Periodontology*).

## 4 DISCUSSION

We investigated whether the polymorphisms linked to serum MMP‐8 associate with periodontitis, periodontal parameters, and saliva concentrations of TCC, S100A8, and S100A12 in a well‐characterized, relatively large sample of middle‐aged or elderly participants. Among the studied polymorphisms of *CFH*, *MMP8*, and *S100A8/S100A9/S100A12* gene region, those located in the *CFH* gene associated with periodontitis, whereas the ones near *S100A9* or in the *MMP8* gene did not. Although the measured saliva concentrations of TCC, S100A8, and S100A12 were significantly higher in periodontitis, the corresponding genetic polymorphisms did not correlate with the saliva levels. Polymorphisms of *S100A9* or *MMP8* did not associate with any periodontal parameters, whereas polymorphisms in *CFH* gene as well as saliva TCC concentrations were associated with clinical and radiographic signs of periodontitis.

The alternative pathway of complement is crucially involved in periodontal dysbiosis and inflammation. It is one of three complement pathways leading to phagocytosis after opsonizing the microbes. The pathway may potentially contribute to ≥80% of total complement activation.^3^ In addition to microbes, potential activators are bacterial virulence factors, membrane polysaccharides, or immunoglobulin A. Studies with several periodontal bacteria, such as *Porphyromonas gingivalis*, *Aggregatibacter actinomycetemcomitans*, *Prevotella intermedia*, and *Tannerella forsythia*, propose that they interact with complement in complex ways that include both inhibitory and stimulatory effects.^2^, ^21^, ^22^ For example, whereas bacterial proteases may cleave C5 to release biologically active C5a, the same proteases readily destroy the C5b component to prevent the generation of TCC.^21^, ^22^ Thus, dysbiotic bacteria in periodontitis have evolved mechanisms to regulate inflammation for their own benefit.^2^ These bacterial species were highly abundant in the saliva samples of the present study, and their amount was associated with moderate to severe periodontitis.^23^

The soluble TCC measured from saliva samples of the present study is not a single molecular species, but a family of multimolecular complexes closely related with each other.^14^ In our study, periodontitis patients had increased levels of saliva TCC, reflecting overall complement activation. The TCC concentration associated with all periodontal parameters examined, ABL, PPD, and BOP, further supporting the idea that excessive complement activation is involved in the pathogenesis of periodontitis and in all steps of the disease progression.

TCC was lower in smokers and TCC associated with periodontitis only in the adjusted regression model in the present study. Thus, smoking may be a relevant confounding factor of saliva TCC determinations. Smoking induces free radicals and oxidative damage and depletes antioxidants, thereby increasing complement activation.^24^ Cigarette smoke can modify C3 for diminished binging to CFH, which leads to activation of the alternative complement pathway.^25^, ^26^ A mouse study showed that “a functional complement alternative pathway” is causative in ocular pathology induced by exposure to cigarette smoke.^24^ However, no published information on the effects of smoking on terminal pathway or TCC was available.

We found that salivary S100A8 and S100A12 were associated with ABL, a high number of periodontal pockets with PPD 4 to 5 mm, and especially with deep pockets (PPD ≥ 6 mm). In addition, they were associated with moderate to severe periodontitis. The associations were strongest for S100A8. Concentrations of S100A8 or S100A12 were not associated with BOP, although in a previous study S100A8/A9 (calprotectin) and S100A12 had an association with BOP and their levels were higher in patients with high modified periodontal inflammatory burden index.^10^ In the same study, the mean level of S100A12 was lower in smokers. Another study showed that S100A8/A9 (calprotectin) levels were significantly higher in both the saliva and serum of patients with periodontitis and salivary S100A8/A9 (calprotectin) had a high diagnostic potential for periodontitis (receiver operating characteristic curve [ROC] = 0.86).^11^ In a recent study, it was discovered that salivary S100A8/A9 (calprotectin) was higher in gingivitis patients compared to healthy, and higher in periodontitis patients compared to healthy or gingivitis patients.^12^ Salivary S100A12 was also elevated in both periodontitis and gingivitis groups compared to control groups, but no significant change was found in the levels of S100A8/A9 (calprotectin) and S100A12 after nonsurgical periodontal therapy. Interestingly, periodontitis patients were clustered in two groups with either high or low levels of salivary S100A8/A9 at baseline.^12^ Longitudinal studies are warranted to investigate whether these biomarkers are associated with periodontal disease activity or successful treatment results.

The common, functional polymorphisms of *CFH* analyzed in the present study lead to structural changes of the molecule. Rs800292 allele A (Val62Ile substitution) is located in domain 1, which together with domains two to four regulates alternative pathway activation by binding to C3b. The polymorphism increases this binding and leads to decreased complement activation. In the present study, the 62I variant was associated with less ABL and lower risk of having periodontitis. Rs1061170 allele C (Tyr402His substitution) is located in domain 7, and it changes the structure of the C‐reactive protein‐binding domain of CFH.^5^ This causes complement dysregulation, since binding of CRP to CFH normally down‐modulates alternative pathway amplification and reduces inflammation.^5^ In the carriers of 402H variant, reduced binding of CRP to CFH would thus lead to a less controlled inflammatory reaction and more tissue damage.^5^ Here, the variant 402H of the rs1061170 polymorphism associated with low number of sites with PPD 4 to 5 mm. Thus, these common polymorphisms of CFH were associated with decreased symptoms of periodontitis and lower risk of having periodontitis.

Rs11225395 is located in the intron of *MMP8*. The T allele is associated with enhanced expression of MMP‐8 and lower risk of generalized aggressive periodontitis,^27^ and the TT genotype significantly associated with an increase in serum MMP‐8 concentrations.^28^ In our study, we did not find associations between rs11225395 and periodontal parameters, although individuals that were heterozygous for rs11225395 had a significantly lower number of pockets with PPD 4 to 5 mm and lower BOP% when the three genotypes were compared. We could hypothesize that both under‐ and overexpression of MMP‐8 may increase the risk of periodontitis, but further studies on the link between periodontitis and rs11225395 are needed for any stronger conclusions.

Rs1560833 is significantly associated with the expressions of S100A12, S100A8, and S100A9 in whole blood,^6^ and the SNP may thus have an impact on systemic inflammation. However, we did not find any association between this SNP and salivary S100A8, S100A12, MMP‐8, or TCC. Saliva reflects mainly local inflammation of the oral cavity and regulation of S100s at protein expression level may not have an effect on oral inflammation. However, it is possible that increased release of these proteins might be implicated in periodontal pathogenesis.

Limitations of the study include the cohort, which represents symptomatic patients with cardiac problems and indication to coronary angiography. Like other inflammatory responses, complement is importantly involved in the pathogenesis of CAD,^29^ although the *CFH* SNPs did not associate with CAD in the present study. Residual confounding cannot be ruled out due to the shared risk factors between CAD and periodontitis, such as smoking, age, male sex, diabetes, and low socioeconomic status. There are no published data on the effect of freezing and thawing on the concentrations of measured proteins. However, all samples received similar freezing and thawing cycles. Excessive complement activation has been associated also with several diseases and pathologies whose association with periodontitis has been suggested, such as Alzheimer's disease,^30^ rheumatoid arthritis,^31^ AMD,^32^ and lung diseases,^33^ involving dysregulations of inflammatory pathways. Since the present study has a cross‐sectional design, conclusions of causality cannot be drawn. However, the cohort also forms a strength of the study, since it is well‐characterized and relatively large.

## 5 CONCLUSION

Our study further supports the observations that any dysregulation of complement may increase the risk of inflammatory disorders, such as periodontitis, and that this is evident both on genetic and expression level. Complement activation is a promising diagnostic and therapeutic target in periodontal diseases.

## CONFLICT OF INTEREST

This study was supported by grants from the Finnish Dental Society Apollonia, Finland; the Selma and Maja‐Lisa Selander Foundation, Finland; and the Academy of Finland (#1340750), Finland. T.S. is the inventor of US patents 5652223, 5736341, 5864632, 6143476, 2017/0023571A1, WO2018/060553A1, 10488415B2, and 2017/0023671A1 and a Japanese patent 2016‐554676. The other authors report no conflicts of interest related to this study.

## AUTHOR CONTRIBUTIONS

Aino Salminen, Milla Pietiäinen, Susanna Paju, Päivi Mäntylä, Kåre Buhlin, and Juha Sinisalo have been involved in data collection. Aino Salminen has performed data analysis. Aino Salminen drafted the manuscript and Milla Pietiäinen, Susanna Paju, Timo Sorsa, Päivi Mäntylä, Kåre Buhlin, Juha Sinisalo, and Pirkko J. Pussinen revised it critically. All authors have contributed to the conception and design of the study, have been involved in data interpretation, and have given final approval of the version to be published.

## Supporting information

Supplementary information

Supplementary information

## Footnotes

1 C___2530382_10 for rs800292, C___9626217_10 for rs1560833, and C___1366493_10 for rs11225395, Applied Biosystems, Thermo Fisher Scientific, Waltham, MA, USA

2 Platinum Superfi PCR Master Mix, Thermo Fisher Scientific, Waltham, MA, USA

3 Mx3000P qPCR System, Agilent, Santa Clara, CA, USA

4 Platinum Superfi PCR Master Mix, Thermo Fisher Scientific, Waltham, MA, USA

5 FastDigest *Tas*I enzyme, Thermo Fisher Scientific, Waltham, MA, USA

6 Human terminal complement complex (TCC) ELISA kit, Hycult Biotech, Uden, The Netherlands

7 S100A8 ELISA Kit (Human), Aviva System Biology, San Diego, CA, USA

8 S100A12 ELISA Kit (Human), Aviva System Biology, San Diego, CA, USA
