# Supplementary material for: Common complement factor H polymorphisms are linked with periodontitis in elderly patients
Source: J Periodontol. 2022 May 4;93(11):1626–34. doi: 10.1002/JPER.22-0005 (PMC10084333; doi:10.1002/JPER.22-0005)
Supplement: Supplementary file 1 — Supplementary information [file JPER-93-1626-s001.docx]

**Supplementary Table 1**. Concentrations of salivary proteins in subgroups of study participants.

|  |  | **S100A8** | **S100A12** | **TCC** | **MMP-8** |
| --- | --- | --- | --- | --- | --- |
|  |  | **median (IQR)** | | | |
| Smoking | no | 25.7 (40.4) | 311 (374) | 168 (518) | 917 (1070) |
|  | yes | 25.5 (50.9) | 341 (377) | 0 (229) | 1086 (1268) |
|  |  | p = 0.90 | p = 0.84 | **p < 0.001** | p = 0.53 |
| Diabetes | no | 24.2 (33.7) | 302 (333) | 135 (431) | 898 (1133) |
|  | yes | 39.5 (57.9) | 367 (510) | 200 (619) | 1050 (1064) |
|  |  | **p < 0.01** | **p < 0.01** | p = 0.23 | p = 0.14 |
| CAD | no | 21.7 (29.8) | 302 (362) | 156 (466) | 821 (998) |
|  | yes | 28.7 (47.7) | 326 (359) | 147 (484) | 1003 (1131) |
|  |  | p = 0.20 | p = 0.70 | p = 0.77 | p = 0.42 |

Concentration for S100A8: ng/ml, S100A12: pg/ml, TCC: mAu/ml, MMP-8: ng/ml. Mann Whitney test. Statistically significant p-values are indicated by bold face. IQR, interquartile range; CAD, coronary artery disease.
