# Supplementary material for: Common complement factor H polymorphisms are linked with periodontitis in elderly patients
Source: J Periodontol. 2022 May 4;93(11):1626–34. doi: 10.1002/JPER.22-0005 (PMC10084333; doi:10.1002/JPER.22-0005)
Supplement: Supplementary file 2 — Supplementary information [file JPER-93-1626-s002.docx]

**Supplementary Table 2.** Associations of SNPs with salivary S100A8, S100A12, TCC, and MMP-8 concentrations. Linear regression adjusted for age, sex, smoking, diabetes, and the number of teeth. Additional model for SNP genotype.

|  |  | **rs1560833** | **rs11225395** | **rs800292** | **rs1061170** |
| --- | --- | --- | --- | --- | --- |
|  | | **B (SD)** | | | |
| S100A8 | | -0.39 (3.39) | -3.48 (6.77) | 4.79 (3.33) | -4.69 (3.08) |
|  |  | p = 0.91 | p = 0.29 | p = 0.15 | p = 0.13 |
|  |  |  |  |  |  |
| S100A12 | | -9.30 (41.3) | -26.6 (39.6) | -15.4 (41.9) | 10.5 (37.7) |
|  |  | p = 0.82 | p = 0.50 | p = 0.71 | p = 0.78 |
|  |  |  |  |  |  |
| TCC |  | -2.90 (142) | -77.2 (67.6) | 5.75 (69.4) | 45.6 (63.9) |
|  |  | p = 0.97 | p = 0.25 | p = 0.93 | p = 0.48 |
|  |  |  |  |  |  |
| MMP-8 |  | -18.2 (40.9) | -2.60 (39.8) | 2.71 (40.6) | 4.91 (37.3) |
|  |  | p = 0.66 | p = 0.95 | p = 0.95 | p = 0.90 |

B, regression coefficient; SD, standard deviation.
